# Sacrococcygeal Teratoma With a Unique Gastrointestinal Presentation of Necrotizing Enterocolitis

**DOI:** 10.7759/cureus.72904

**Published:** 2024-11-02

**Authors:** Animesh Sahu, Richie Dalai, Saurav Srivastava, Bhabesh K Chowdhary, Madhuri Kumari

**Affiliations:** 1 Department of Neonatology, All India Institute of Medical Sciences, Patna, Patna, IND; 2 Department of Pediatric Surgery, All India Institute of Medical Sciences, Patna, Patna, IND; 3 Department of Radiology, All India Institute of Medical Sciences, Patna, Patna, IND

**Keywords:** necrotizing enterocolitis, neonatal intensive care, neonate, pediatric surgery, sacrococcygeal teratoma

## Abstract

A neonate with an antenatally detected mass in the coccygeal region experienced sudden abdominal worsening, with abdominal distension and bloody aspirates on day 4 of life. The abdominal radiograph revealed pneumatosis intestinalis (necrotizing enterocolitis stage IIa), requiring discontinuation of feeds and initiation of intravenous antibiotics. Computed tomography of the mass and abdomen showed that the coccygeal mass had an intra-abdominal extension, pushing the bowel loops and likely representing a Type II sacrococcygeal teratoma. Given the lesion's large size and vascularity, we hypothesize that chronic bowel compression with vascular steal may have contributed to gut ischemia, stasis, and subsequent necrotizing enterocolitis. The mass was excised, and the neonate subsequently remained asymptomatic. This is the first reported case of necrotizing enterocolitis in the setting of a sacrococcygeal teratoma in a neonate. This case also underscores the importance of vigilant monitoring of neonates with sacrococcygeal teratoma for complications arising from its mass effect apart from routine hemodynamic monitoring.

## Introduction

Sacrococcygeal teratomas (SCTs) are the most common germ cell malignancies in neonates and infants, with a reported incidence of 1 per 35,000-40,000 live births. They are three times more common in females than in males and most commonly arise from the base of the coccyx. Though mostly benign, around 1-2% exhibit malignancy. Fetuses with large SCTs can develop high-output cardiac failure, hydrops, and even death due to the high metabolic demands. Additionally, the redirection of systemic blood volume into the tumor may occur, caused by its rapid growth and low-resistance blood vessels, a phenomenon termed vascular steal. A large SCT can exert a significant mass effect on adjacent structures and may result in various comorbidities such as obstructive hydronephrosis, anterior displacement of the anus and rectum, proximal bowel distension, and hip dislocation [1]. The mainstay of treatment is complete surgical resection, with recurrence being infrequent [2]. Large type III SCTs often require extensive resection and carry a high risk for poor functional outcomes [3]. Therefore, holistic monitoring of any neonate with SCT, for all associated symptoms, along with timely surgical management, is key to a good outcome. Here we present a case of a large type II SCT in a neonate and discuss the consequences of its compressive symptoms, which may have contributed to the development of necrotizing enterocolitis (NEC).

## Case presentation

A 31-year-old second gravida female presented at 33 weeks and 3 days of gestation and underwent an emergency cesarean section due to fetal distress. Her early pregnancy was uncomplicated; however, a routine 32-week sonogram revealed a highly vascular mass in the coccygeal region, measuring approximately 7 × 7 × 6 cm. The neonate weighed 2120 grams at birth and had a delayed cry, requiring positive pressure ventilation for 30 seconds. Following this, the neonate showed good spontaneous efforts and had an APGAR (Appearance, Pulse, Grimace, Activity, and Respiration) score of 7 at 5 minutes of life. However, the neonate developed respiratory distress with moderate retractions and grunting, requiring continuous positive airway pressure of 5 cm water and a fraction of inspired oxygen (fiO₂) of 21%. Venous blood gas at 1 hour of life showed a pH of 7.39 and a base deficit of -8.9, with no features suggestive of hypoxic-ischemic encephalopathy. Respiratory distress gradually improved, and orogastric tube feeds of expressed breast milk and donor human milk were initiated at 6 hours of life.

**Figure 1:**
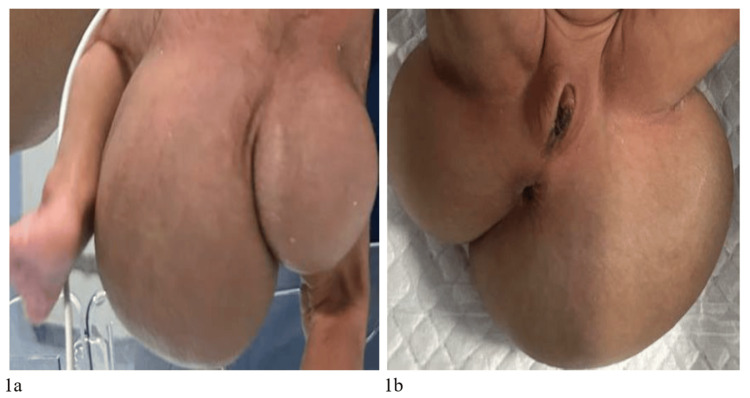
Neonate with a huge sacrococcygeal teratoma: (a) posterior view; (b) anterior view.

On examination, the neonate had a soft, fluctuant swelling measuring 6 × 7 × 5 cm in the perivulvar region, extending from the sacral area and involving the genitals and buttocks (Figure 1a, b). A bedside ultrasonogram of the lesion suggested a heterogeneous lesion, 10 × 4 cm in size, in the right labial area with cystic and echogenic content and intervening vessels showing flow on Doppler. Further imaging was advised post-stabilization to delineate the lesion contents.

**Figure 2:**
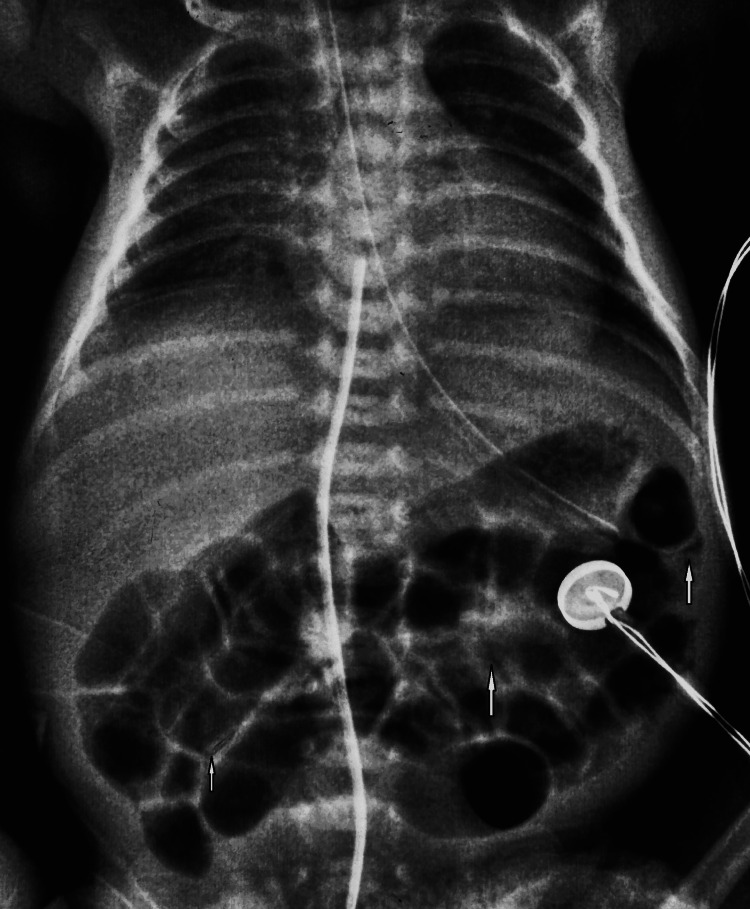
Abdominal radiograph showing intramural gas or pneumatosis intestinalis represented by white arrows, suggestive of NEC IIa.

On her 4th day of life, the neonate developed feed intolerance with abdominal distension and blood-tinged gastric aspirates. The sepsis screen was negative. An abdominal erect radiograph showed pneumatosis intestinalis in a few loops, consistent with necrotizing enterocolitis stage IIa (NEC IIa) (Figure 2). There was no evidence of portal venous gas or pneumoperitoneum. Oral feeds were discontinued, and she was started on total parenteral nutrition and intravenous antibiotics, piperacillin-tazobactam and amikacin, which were administered for 10 days.

**Figure 3:**
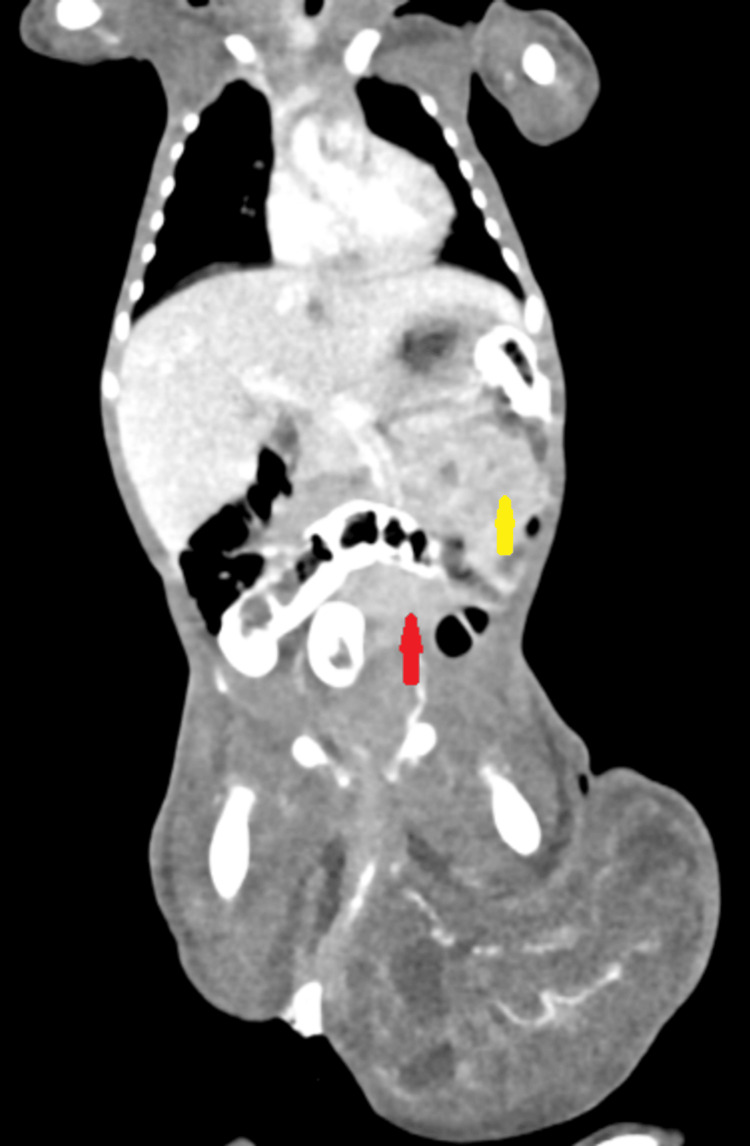
Computed tomography scan, coronal section of the abdomen and pelvis, showing a large solid-cystic mass lesion centered in the coccygeal region with intrapelvic and intra-abdominal extension. Red arrow: Tumor mass with intraabdominal extension. Yellow arrow: Compressed and collapsed small bowel loops.

Feeds were gradually reintroduced on day 10 of postnatal age, and the neonate achieved full feeds by day 14. A computed tomography scan performed post-stabilization revealed a large, well-defined solid-cystic lesion measuring 2.7 × 12.4 × 8.4 cm arising from the coccygeal region with intrapelvic extension. Anteriorly, it displaced the visceral structures, including the bladder and bowel, without mucosal involvement. The lesion had a prominent arterial feeder from the rectal and left internal iliac arteries, supporting the diagnosis of SCT (Figure 3). Additional blood investigations for tumor markers were conducted, as detailed in Table 1.

**Table 1:** Blood investigations of the neonate as a part of sepsis workup and tumor markers. LDH: lactate dehydrogenase, CA 125: cancer antigen 125, HCG: human chorionic gonadotropin, AFP: alpha-fetoprotein, NA: not available.

Day of life	Day 1	Day 5	Day 17	Reference values
Hemoglobin (g/dL)	15.6	14.8	NA	12-20
TLC (cells/µL)	5130	8530	NA	4000-10000
Platelets (cells/µL)	1.9 lakhs	2.6 lakhs	NA	1.5-4.5 lakhs
Blood culture	Sterile	Sterile	NA	-
C-reactive protein (mg/L)	1.2	NA	NA	<10
Urea (mg/dL)	18	NA	NA	13-43
Creatinine (mg/dL)	0.2	NA	NA	0.2-0.9
Thyroid-stimulating hormone (µIU/mL)	6.2	NA	NA	<20
LDH (U/L)	NA	NA	348	135-750
CA 125 (U/mL)	NA	NA	9.8	0-35
HCG (mIU/mL)	NA	NA	2.1	<10
AFP (ng/mL)	NA	NA	>1000	2000-400,000

**Figure 4:**
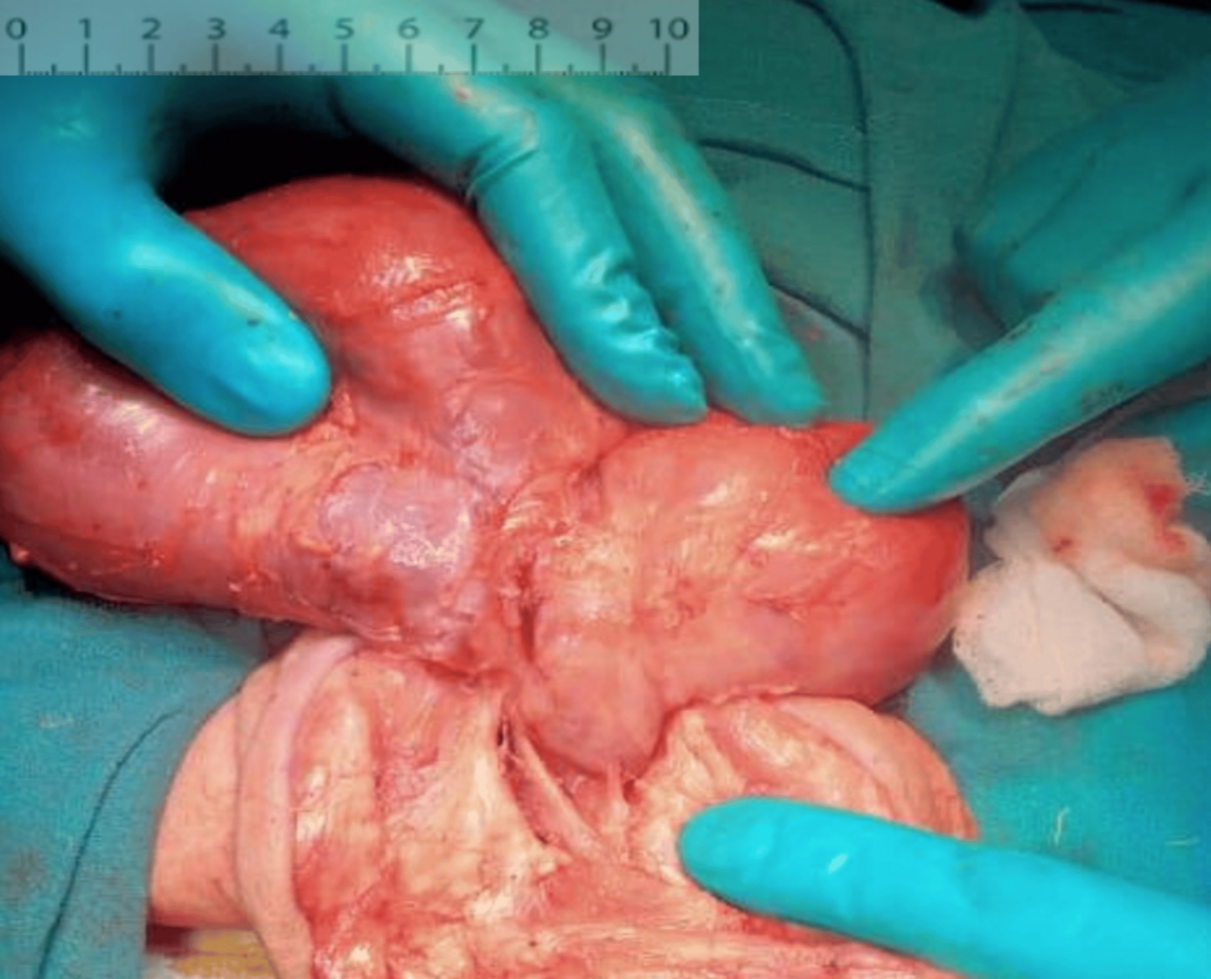
Intraoperative image of the sacrococcygeal teratoma (note the size and vascularity).

Pediatric surgery was consulted, and the mass was excised along with coccygectomy via the perineal route on day 19 of life (Figure 4). Histopathology revealed a 14 × 8 × 4 cm solid-cystic lesion with derivatives from all three germ layers. Post-procedure, the neonate remained hemodynamically stable and was extubated within 24 hours of surgery. Feeds were restarted on postoperative day 2 and progressed to full feeds, which were well tolerated. The neonate was subsequently discharged at four weeks of life.

At follow-up at four months, she is exclusively breastfed, gaining weight adequately, has a healthy suture site, and is neurologically normal.

## Discussion

Large SCTs can lead to bladder and bowel obstruction due to their mass effect [4,5]. Signs and symptoms of these obstructive symptoms should be carefully sought and monitored. Based on the modified Bell staging for NEC, the index case had NEC IIA [6].

NEC is frequently encountered in premature neonates and there are multiple predisposing causes. The common pathway is gut ischemia, stasis, infection, inflammation, and necrosis. In our case, there were no risk factors for sepsis and the sepsis workup was negative. Prematurity is a risk factor for NEC with the age of onset being inversely proportional to postmenstrual age. However, most of these cases occur in neonates who are <1500 grams and <32 weeks gestational age [7]. Etiologies in older neonates usually include congenital heart diseases or anomalies that may lead to intestinal ischemia. Since this case had a massive SCT with intra-abdominal extension, there was likely bowel compression, leading to ischemia. This could have led to stasis, infection, and the subsequent NEC. This lesion was focally adhered to the rectal wall, had significant internal vascularity, and was supplied by left internal iliac and rectal arteries. This may have resulted in bowel ischemia secondary to vascular steal. However, there were no other systemic features of vascular steal in the form of congestive heart failure or hydrops [2,8].

The gastrointestinal system manifestations due to the SCT reported so far include abdominal distension secondary to intestinal obstruction, constipation, and fecal incontinence [9,10]. To the best of our knowledge, NEC in the setting of an SCT has not been reported till now. However, one cannot conclude causality till further observational studies are available. This case underscores the importance of evaluation and monitoring of neonates with SCT for complications leading to compression, displacement of surrounding organs, and possible predisposition to NEC.

## Conclusions

Neonates with an SCT require close monitoring for obstructive symptoms. This case describes NEC stage II in the setting of an SCT in a neonate. Further observational studies are needed to determine if this is a true association.
